# Improved urine DNA methylation panel for early bladder cancer detection

**DOI:** 10.1186/s12885-022-09268-y

**Published:** 2022-03-03

**Authors:** Qixun Fang, Xu Zhang, Qing Nie, Jianqiang Hu, Shujun Zhou, Chaojun Wang

**Affiliations:** 1 Yaneng Bioscience, Co., Ltd, Shenzhen, 518100 China; 2 South China University of Technology, Guangzhou, 510641 China; 3 Department of Urology, the First Affiliated Hospital, Zhejiang University School of Medicine, Hangzhou, 310003 China

**Keywords:** Urine, Bladder cancer, Biomarkers, Methylation, Diagnosis, RRBS, qMSP

## Abstract

**Background:**

Bladder cancer is one of the most common malignancies but the corresponding diagnostic methods are either invasive or limited in specificity and/or sensitivity. This study aimed to develop a urine-based methylation panel for bladder cancer detection by improving published panels and validate performance of the new panel with clinical samples.

**Methods:**

Related researches were reviewed and 19 potential panels were selected. RRBS was performed on a cohort with 45 samples to reassess these panels and a new panel inherited best markers was developed. The new panel was applied with qMSP platform to 33 samples from the RRBS cohort and the results were compared to those of RRBS. Lastly, another larger cohort with 207 samples was used to validate new panel performance with qMSP.

**Results:**

Three biomarkers (PCDH17, POU4F2 and PENK) were selected to construct a new panel P3. P3 panel achieved 100% specificity and 71% sensitivity with RRBS in corresponding cohort and then showed a better performance of 100% specificity and 84% sensitivity with qMSP platforms in a balanced cohort. When validated with 207-sample cohort, P3 with qMSP showed a performance of 97% specificity and 87% sensitivity which was modestly improved compared to the panels it derided from.

**Conclusions:**

Overall, the P3 panel achieved relatively high sensitivity and accuracy in bladder cancer detection.

**Supplementary Information:**

The online version contains supplementary material available at 10.1186/s12885-022-09268-y.

## Background

Bladder cancer (BC) is known as one of the most common malignancies in the world [1]. It was reported that 75% of the primary tumors were in non-muscle-invasive Ta or T1 stage while others show bladder muscle invasion in stages T2–4. Clinically, stage Ta BC was characterized by frequent recurrence after resection in up to 60% of patients [2]. Typically, within 8 to 10 years, one or more tumors would appear each year and 25% of them would eventually develop into an aggressive invasive phenotype [3].

Currently, cystoscopy/biopsy is the gold standard for the BC detection of suspicious lesions. Unfortunately, this expensive, invasive and painful procedure would miss 10 to 40% of malignancies including up to 15% of the papillary carcinoma and up to 30% of the flat recurrences. Urine sediment was proposed as samples for non-invasive detection methods. However, although urine cytology possesses a high specificity, it lacks of sensitivity, particularly in low-risk tumors [4]. The applications of nuclear matrix protein 22 (NMP-22), bladder tumor antigen and UroVysion FISH were proposed as complement to improve cytology sensitivity, but they were rarely adopted in clinical practice due to unsatisfying performance [1, 5, 6].

Many researchers were committed to develop better markers for BC diagnosis and prognosis. DNA methylation, which plays an important role in transcription regulation [7, 8], had been found to be chemically stable and quantifiable in high precision, making it a competitive candidate within tumor markers [9, 10]. Inactivation of tumor suppressor genes by local DNA hypermethylation and activation of suppressed genes by global DNA hypomethylation had been both observed in bladder tumors [11–13]. Further studies also demonstrated that methylation changes found in urine sediments resemble those found in tumor tissues [14–16].

In last few decades, an increasing number of DNA methylation markers had been developed with different techniques such as MS-MLPA, quantitative methylation-specific PCR (qMSP) and pyrosequencing for BC detection. However, in most cases, the performance of marker panels was not satisfying. Thus, it is still urgent to seek a reliable DNA methylation marker set operating on a low-cost platform with high sensitivity and specificity for BC detection. Reduced representation bisulfite sequencing (RRBS) is a technique for genome-wide methylation profiles analysis with single nucleotide precision. One of the main goals for RRBS study is to discover differentially methylated regions (DMRs) between different samples, which makes it a suitable tool for methylation biomarker screening and assessing.

In this study, we aimed to develop a reliable DNA methylation panel for early BC detection in clinical practise. We composed an improved urine DNA methylation panel based on other published methylation panels. This newly proposed panel was compared with published panels using RRBS and further applied to clinical diagnosis with qMSP.

## Methods

### Study design

**Fig. 1:**
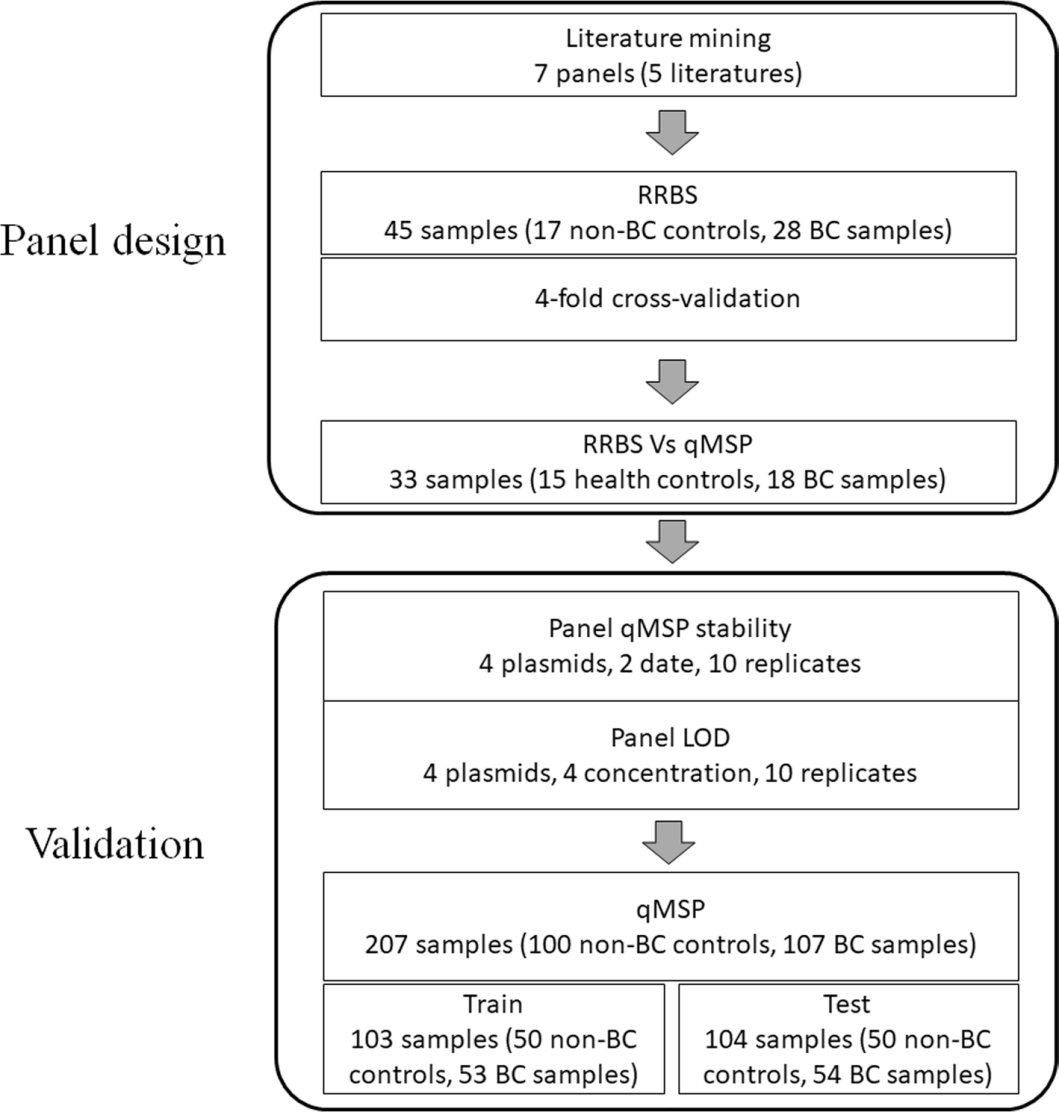
Outline of study design. The performance of reviewed panels was assessed using RRBS with 28 BC patient samples with 17 non-BC controls (15 healthy samples and 2 patient samples with non-BC bladder diseases). After a new panel was composed, for comparison with RRBS results, 18 BC samples and 15 healthy samples from the RRBS cohort were tested by the new panel with qMSP. The new panel was then analytically validated with synthesized plasmids. Another cohort with 107 BC patients, 24 non-BC patients with other bladder diseases and 76 healthy controls was used to validate new panel’s performance. BC: bladder cancer, qMSP: quantitative methylation-specific PCR, LOD: limit of detection

In this study, panel development went through two stages: panel design and validation. In panel design stage, a new panel was composed based on RRBS assessment of a collection of methylation panels for BC detection proposed by literatures. RRBS results with the new panel were then compared with results of qMSP. In validation phase, analytical validation with synthesized plasmids and performance validation with another larger clinical cohort were conducted for the new panel. The outline of this study is illustrated in Fig. 1.

### Patients and sample collection

All patients with BC were clinically confirmed with multiple methods including but not limited to cystoscopy, cytology and surgery. All samples were collected from volunteers of both sex above 18 years old. BC patients included were not intentionally balanced on any aspect and patients with prior chemotherapeutic treatment could be included.

The fresh urine samples were obtained from participates via the First Affiliated Hospital, Zhejiang University School of Medicine. Cell pellets were centrifuged and kept frozen until used for DNA extraction. Clinical, demographic, and pathological data were collected for all patients. Informed consent was obtained from all participants. This research was approved by Zhejiang University Institutional Review Board including that all methods were performed in accordance with the relevant guidelines and regulations.

### DNA extraction

DNA from the urinary cell pellets was extracted using QIAamp DNA Mini Kit (Qiagen) according to the manufacturer’s instructions. DNA quality and quantity were assessed using a NanoDrop2000 (Thermo Scientific) spectrophotometer and 1% agarose gel electrophoresis.

### RRBS library construction

100 ng genomic DNA per sample was used to construct RRBS libraries. Genomic DNA was digested overnight with MspI restriction enzyme (recognition site C^CGG, NEB, Ipswich, MA, USA) at 37 °C. After purification, digested DNA was treated with a mix of T4 DNA polymerase, Klenow Fragments and T4 polynucleotide kinase to repair, blunt and phosphorylate ends. The mixture DNA fragments were subsequently 3′ adenylated using Klenow Fragments (3′-5′ exo-) then ligated to adaptors with 5′-methylcytosine substituting cytosine using T4 DNA Ligase. Bisulfite conversion was performed with a ZYMO EZ DNA Methylation-Gold Kit (ZYMO, Irvine, CA, USA) and lambda DNA was added as a conversion marker. The final libraries were generated by 13 cycles PCR amplification and then quantified by an Agilent 2100 Bioanalyzer (Agilent Technologies). Sequencing were performed on an Illumina Hiseq platform with a pair-end 300 cycles setting.

### Data analysis

Raw sequencing data were processed by the Illumina base-calling pipeline. Low-quality reads that contained more than 30% ‘N’s or over 10% low-quality calls (quality value < 20) were removed. Adapter contamination was removed by cutadapt (version 1.9) [17]. The clean reads were aligned to the reference genome hg19 using BSMAP (version 2.73) [18]. Conversion ratio was calculated on lambda DNA and samples with a conversion ratio lower than 99% would be considered unqualified for further analysis and corresponding libraries would rebuilt and sequenced. Only uniquely aligned reads containing the *Msp* I enzyme digestion sites were used for further analysis. Only CpG sites with sequencing depths ≥5 were selected as candidate sites. After bisulfite treatment, cytosines were read as “T” if unmethylated or as “C” if methylated. Methylation level of the sample was defined as the ratio of number of “C”s and the sequencing depth of the site. Differential methylation analysis on these sites were performed with ANOVA (analysis of variance) and the acquired *p*-values were adjusted to Q-values with Benjamini-Hochberg method [19]. Differential methylation sites (DMSs) were defined as sites with a Q-value lower than 0.05.

### Performance verification with RRBS results

To verify panel performance, DMS related to marker genes (within 5000 bp to gene region) were extracted from RRBS differential analysis. These DMS were grouped by individual panel and used as sample features for model training and sample classification. Support vector machine (SVM) with radial basis function (RBF) kernel was applied as the main classification model and the panel performance was evaluated in 4-fold/3-fold cross validation manners where the cohort was divided into 4/3 groups with equal cancer/control proportion and reserve one group for testing model performance with other groups used for model training. The final panel performance was obtained by averaging performance estimation of all 4/3 tests. Confusion matrixes and Receiver Operating Characteristic (ROC) curves were preserved for further performance analysis.

### Methylation measuring with qMSP

DNA samples were treated by EZ DNA Methylation-Gold™ Kit (Zymo Research). The bisulfite modification reaction was executed by 96-Well GeneAmp PCR System 9700 (Applied biosystems) with the 150 μl mixtures containing 130 μl CT conversion reagent (Zymo Research) and 200 ng DNA template. The condition of the reaction was configured to 98 °C for 10 min followed by 64 °C for 2.5 h and then on hold at 4 °C. Each DNA sample was then purified by 20 μl of M-Elution Buffer (Zymo Research) and stored at − 20 °C before use.

Primers and probes (Table 1) of candidate biomarkers (PCDH17, POU4F2, and PENK) and β-actin (reference gene) were used in the qMSP assay. Each reaction mixture (25 μl in total) was consisted with 5 μl of 5X probe qPCR Buffer (Tiagen), 2.5 μl of 10X HA Buffer, 2 μl of dNTP (2.5 mM), 9.5 μl primer and probe mix, 0.75 μl HA Taq, 0.25 μl Taq-Antibody, and 5 μl of DNA template. The reactions were performed on 7500 Real-Time PCR System (Applied Biosystems) where samples were pre-incubated for 5 min at 95 °C, and then amplified for 48 cycles at 95 °C for 15 s and 60 °C for 40 s. Fluorescence signals were measured at the end of each extension step at 60 °C. The measurements were done in triplicate and mean Ct values were used as final Ct values. According to [20], the site methylation rate (SMR) referencing ACTB can be calculated as:

$$SMR={2}^{\left[ Ct(ACTB)- Ct\right]}\times 100$$

**Table 1:** Primer and probe sequences of P3 markers

Gene	Forward sequence	Reverse sequence	Probe sequence
β-Actin	GGAGGTAGGGAGTATATAGGTTG	CACACAATAACAAACACAAATTCAC	AAACTTACTAAACCTCCTCCATCACCACCC
PCDH17	CGGGTGTTGGAGAATTTCG	CGCGATCGATACGCTACTTA	CCGCTATCTACGTCCACGTCCAACA
POU4F2	AAGGGTTGTGCGAAGTTG	AACGCGTAACCGAAATCA	CGTACAAAATCCGAAAACGACGACGAA
PENK	GGTTGTTGTTGTTCGGTTTC	CGACCGAACGCACTAAAC	AACTACACGTCGCGCAATCCTAACTACAT

where Ct (ACTB) and Ct are Ct values of ACTB reference and the target marker respectively. Thus, to simplify, *Ct* − *Ct*(*ACTB*) as ΔCt was used as a proxy of the SMR. However, in this case, ΔCt increases with the decrease of SMR and the correlation between is logarithmic.

### Cancer detection with qMSP results

QMSP data was all analysed by Random Forest (RF) model with 100 decision trees whose maximum depth was set to 5 when three markers were involved and 4 otherwise. The cohort was first split into training set and testing set in a random manner while maintaining identical cancer/control ratio. After training and testing, prediction sequence, sensitivity, specificity, accuracy, AUC and ROC curves were calculated and exported as results.

### Reproducibility and limit of detection (LOD) study

Investigation on reproducibility and LOD requires stable standard samples with certain methylation ratio. Since completely methylated sequences does not change in bisulfide conversion, probe sequences (Table 1) were synthesized for each marker, including ATCB, to simulate completely methylated sequences and standard samples were obtained by mixing plasmids carrying synthesized sequences in different concentration (details shown on supplement Table **1**). These plasmids were generated by cloning sequences to certain vectors. The synthesized ACTB probe sequences were ligated into pGSI/Amp vectors via SmaI sites to construct ACTB plasmids, while other plasmids were built with probe sequences ligated into pUC-GW-Kan/Amp vectors via the EcoRV sites. For reproducibility study, testing with standard samples were repeated 10 times on two separate days with same protocol. For LOD study, standard samples with single plasmids in 4 concentrations were measured.

## Results

### Novel panel design with relevant literature

Methylation based BC detection panels on urine sedimentation samples with potential high performance suggested by published literature were selected. Overall, panels with more markers were more sensitive but less specific toward BCs (Table 2). Nonetheless, a panel consisting only PCDH17 and POU4F2 but reported as one of the best performing panels reviewed in terms of sensitivity and specificity attracted our interest. Additionally, although only a few genes were selected by multiple panels, the two markers in this panel were completely inherited by another well performing panel. Panel performance was evaluated under different circumstances, namely different platforms and cohorts, and not always available. Therefore, performance reassessment on these panels with the same technique and cohort was required for a more comprehensive comparison.

**Table 2:** Reviewed panels for bladder cancer detection

ID	Gene Panel	Method	Published results		RRBS results			ref
			SP	SN	SP	SN	AC	
1	TIMP3,APC,CDKN2A,MLH1,ATM,RARB,CDKN2B,HIC1,CHFR,BRCA1,CASP8,CDKN1B,PTEN,BRCA2,CD44,RASSF1,DAPK1,FHIT,VHL,ESR1,TP73,IGSF4,GSTP1,CDH13	MS-MLPA	–	–	1.00	0.83	0.89	[17]
2	HIC1,RASSF1,GSTP1	MS-MLPA	0.66	0.78	0.91	0.67	0.76	[17]
3	HOXA9,ISL1	qMSP	0.91	0.44	0.99	0.71	0.78	[18]
4	PCDH17,POU4F2	qMSP	0.94	0.90	1.00	0.73	0.81	[19]
5	E2F3,CCND1,UTP6,CDADC1,SLC35E3,METRNL,TPCN2,NACC2,VGLL4,PTEN	metadata	–	–	0.98	0.63	0.73	[20]
6	CDH13,CFTR,NID2,SALL3,TMEFF2,TWIST1,VIM2	pyrosequencing	–	–	1.00	0.63	0.76	[21]
7	CFTR,SALL3,TWIST1	pyrosequencing	0.31	0.90	1.00	0.65	0.77	[21]
8	SOX1,TJP2,MYOD,HOXA9_1,HOXA9_2,VAMP8,CASP8,SPP1,IFNG,CAPG,HLADPA1,RIPK3	pyrosequencing	1.00	1.00	1.00	0.74	0.84	[21]
9	ZNF671,SFRP1,IRF8	qMSP	0.84	0.96	1.00	0.59	0.74	[22]
10	TWIST1,NID2	MSP	0.93	0.96	1.00	0.61	0.76	[23]
11	VIM,TMEFF2,GDF15	qMSP	1.00	0.94	1.00	0.54	0.71	[24]
12	VIM,TMEFF2,GDF15,HSPA2	qMSP	1.00	0.94	1.00	0.54	0.71	[24]
13	SALL3,CFTR,ABCC6,HPR1,RASSF1A,MT1A,ALX4,CDH13,RPRM,MINT1,BRCA1	MSP	0.87	0.92	1.00	0.73	0.83	[25]
14	SALL3,CFTR,MT1A,HPP1,ABCC6,RASSF1A,CDH13,RPRM,MINT1,BRCA1,SFRP1	MSP	0.73	0.92	1.00	0.72	0.83	[26]
15	SALL3,CFTR,MT1A,HPP1,ABCC6,RASSF1A,CDH13,RPRM,MINT1,BRCA1	MSP	0.80	0.90	1.00	0.70	0.81	[26]
16	p14ARF,p16INK4A,RASSF1A,DAPK,APC	MSP	–	0.91	1.00	0.64	0.77	[27]
17	RARβ,DAPK,CDH1,p16	MSP	0.76	0.91	1.00	0.66	0.79	[28]
18	HOXA9,PCDH17,POU4F2,ONECUT2	qMSP	0.73	0.91	1.00	0.71	0.82	[29]
19	PENK	qMSP	0.88	0.89	0.96	0.68	0.78	#
P3	PCDH17,POU4F2,PENK	–	–	–	1.00	0.71	0.82	–

*SP* specificity, *SN* sensitivity, *AC* accuracy. # Biomarker reported by Genomictree: http://www.genomictree.com/ko/index.asp

### Panel performance analysis with RRBS on clinical samples

**Fig. 2:**
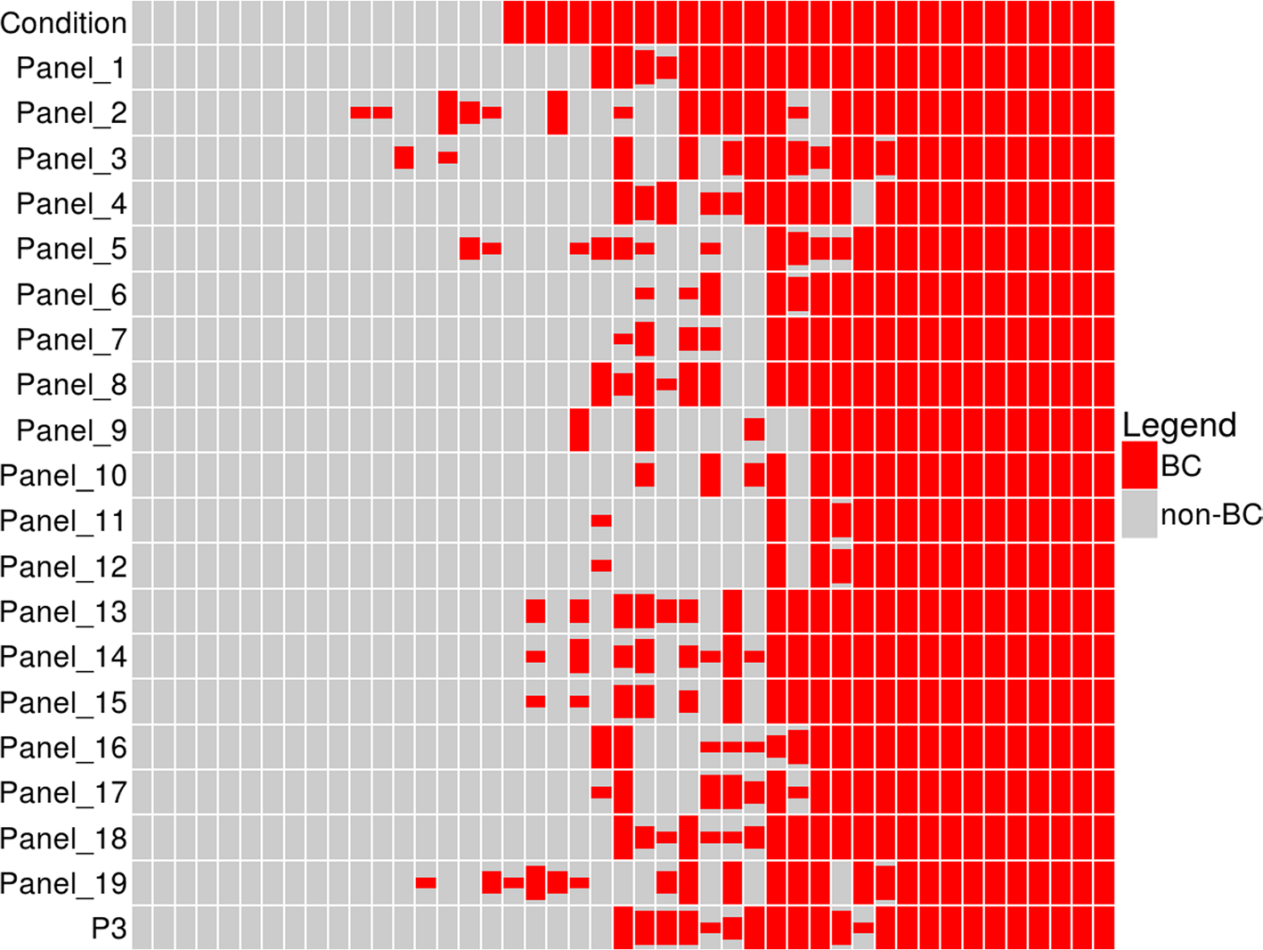
Bladder cancer predictions of reviewed panel and P3 panel. BCs were represented by red blocks and non-BCs by grey blocks. The condition row presents the true status of samples while others display predictions made by panels. A 4-fold cross-validation process was applied so that 4 predictions were made for every sample by every panel. The high of red blocks represents the proportion of the sample being predicted as BC by the corresponding panel in the cross-validation process

RRBS was performed on clinical samples from 45 participates including 28 BC patients, 15 healthy volunteers and 2 patients with other bladder diseases (Table 3) to reassess panels’ performance for BC detection. The specificity, sensitivity and accuracy were generated by averaging corresponding figures in a 4-fold cross validation process with support vector machine (SVM) models. Larsen et al. pointed out that large variability of marker performance can be observed across studies [30] and this statement were confirmed by our reassessment results (Table 2). This variability could be the result of different cohort, platforms and prediction model used. Compared to the published figures, higher specificity was achieved by RRBS in all panels probably due to imbalanced sample class or model bias. Although the detection specificity was generally high, certain BCs were missed by most panels (Fig. 2) suggesting defeats in sensitivity for detecting BC samples with methylation profiles less distinct from those of health samples.

**Table 3:** Clinicopathological and demographical information of involved population

	Verification phase (RRBS)	Validation phase (qMSP)
Sample number	45	207
BC patient	28	107
Non-BC control	17	100
Among patients		
Non-BC		
IMT	2	0
Inflammation	0	19
Bladder stone	0	4
Benign tumor	0	1
BC		
Age range	48–92	29–92
Mean age	67.9	67.2
Stage		
Ta	11	28
T1	6	41
T2	5	23
T3	4	6
T4	2	9
MIBC	13	54
NMIBC	10	53
High grade	18	58
Low grade	10	49
Primary	18	72
Recurrence	10	35

* *IMT* Inflammatory myofibroblastic tumor, *MIBC* Muscle invasive bladder cancer, *NMIBC* Non-muscle invasive bladder cancer. Pathological details were unclear or lost for some samples, these samples were excluded in relative analyses

Best overall performance was achieved by a panel with 24 markers from Casadio et al. [31] with highest scores in all aspects. Most of the investigated panels containing no more than 5 genes only achieved an accuracy lower than 0.8, except the 2-marker panel from Wang et al. [32] and another panel inherits its two markers which managed to achieve results comparable to panels with most gene markers. Apart from these two panels, PENK marker also stood out by achieving a performance close to big panels and slightly surpassed Wang’s panel. Moreover, the PENK marker alone successfully detected BCs that rarely detected by other panels, including these with more than 5 genes (Fig. 2). Therefore, for better performance the three gene markers mentioned above were combined as a new P3 panel but RRBS results showed no substantial improvement.

### Performance comparison of RRBS and qMSP

Although P3 panel was unremarkable in performance measured by RRBS, it was applied to qMSP for further evaluation. Compared to RRBS, different error would be introduced by qMSP but it is a more widely used low-cost methylation measuring platform in clinical practise. 18 BC samples and 15 healthy samples from the cohort tested by RRBS were tested by qMSP in a 3-fold cross validation configuration with SVM instead of 4-fold due to the decrease of sample number. Area under ROC curve (AUC) was also calculated to estimate detection power. The results presented in Table 4 showed qMSP possess higher sensitivity with identical specificity leading to higher accuracy and AUC compared to RRBS. This could be attributed to crucial loci selection by qMSP probes since more near gene loci were included and weighted equally in RRBS detection.

**Table 4:** Performance comparison of RRBS and qMSP in BC detection

METHOD	SP	SN	AC	AUC
RRBS	1.00	0.71	0.84	0.99
QMSP	1.00	0.84	0.92	0.96

*SP* specificity, *SN* sensitivity, *AC* accuracy, *AUC* Area Under ROC curve.

### Performance validation on qMSP platform

#### Analytical validation on synthetic plasmids

For understanding the stability of panel performance, the reproducibility and LOD were investigated. Plasmids were synthesized for each marker, including ATCB, and mixed in certain concentration to compose standard samples. All analytical tests were repeated 10 times where intragroup and intergroup difference was calculated. Detail results can be found in supplement Table 2 and 3. In summary, the panel produced stable results within intended Ct value range.

#### Performance validation with clinical samples

The cohort used in the technique comparison mentioned in the previous section was extended to a larger cohort with 107 BC samples and 100 non-BC samples (76 healthy and 24 with other bladder diseases) (Table 3) for further P3 panel performance validation. RF model, similar to the SVM model, is a model used to classify samples based on certain features. It was applied instead of SVM for BC/non-BC classification as higher accuracy could be achieved with RF when importance is properly distributed to features and overfitting is avoided by adjusting model parameters correlated to number of feature and sample. SVM models were used in previous RRBS detection due to uncertain feature number across panels. The RF model was trained with 50% of the cohort and test with the rest 50% then the entire cohort was included when specificity, sensitivity, accuracy and AUC were calculated.

**Fig. 3:**
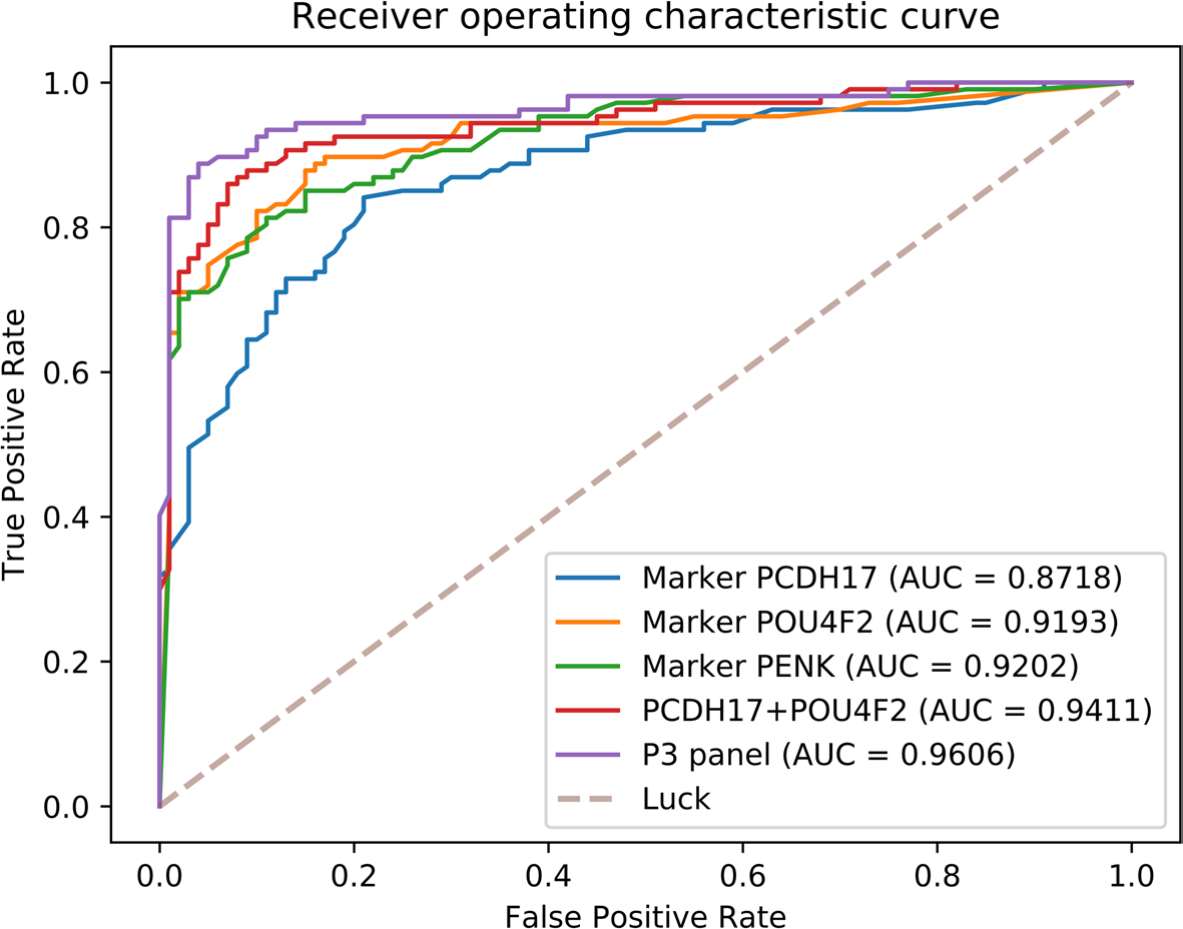
Receiver Operating Characteristic (ROC) curve of individual marker and combined P3 panel

Compared to previous results (Table 4**)**, the P3 panel performed with high consistency in all aspects (see Table 5**and** Fig. 3 for details). Wang’s panel and the PENK marker were also assessed with this cohort and diverse changes in their performances were observed in comparison with RRBS results (Table 2). Only subtle performance change was observed in Wang’s panel while the sensitivity of marker PENK was significantly higher, consequently facilitated improvement observed in Table 4 and contribute to the P3 panel performance which exceeded its parent panels in every aspect. However, this improvement achieved by P3 panel was only moderate.

**Table 5:** Performance of individual marker and p3 panel in BC detection

Gene	SP	SN	AC	AUC
PCDH17	0.83	0.74	0.78	0.87
POU4F2	0.90	0.80	0.85	0.92
PENK	0.93	0.76	0.84	0.92
PCDH17+ POU4F2	0.95	0.78	0.86	0.94
P3	0.97	0.87	0.92	0.96

*SP* specificity, *SN* Sensitivity, *AC* Accuracy, *AUC* Area under ROC curve.

### Panel potential in BC subtype detection

Given proper panels, non-invasive detection methods allow continuous monitoring BC status such as being muscle invasive/non-muscle invasive (MIBC/NMIBC), high/low grade, early/late stage and primary/recurrents. Therefore, we analysed P3 panel performance on BC subtype detection with a train-test configuration (find supplement Table 4 for results). Yet missing status details further shrank the size of valid cohort which was not initially large, preventing reliable conclusion to be drawn. Moreover, notable potential for detecting any subtype had not be observed with P3 panel at this stage.

## Discussion

At the panel development stage, BC detection with RRBS which measures gene methylation by averaging methylation ratio of related locus was conducted and compared to detection with qMSP which associates gene methylation with methylation of certain short sequences. This possess revealed limitations in current panel-base detection methods which might inspire further improvement.

### Number of biomarker limits detection performance

The facts that RRBS detection performance improves with marker number and certain BCs were only detected by one specific panel suggested considerable inter-individual methylation heterogeneity of BCs. The results indicated BC specific methylations mostly converge to a few genes but remarkable distinction occurs due to unclear mechanisms involving extra genes. Similar heterogeneity was also discovered on Ewing sarcoma by Sheffield et al. [33], who had failed in attempt to align EWS-FLI1 fusion related heterogeneity characteristics with disease subtypes. Correlations between methylation heterogeneity and disease subtypes might be identified when including more related genes. The small cohort sequenced in this study was insufficient for investigating these correlations but unknown regulations within BCs might be unveiled when sufficient data is available. Another cause of such heterogeneity could be the aberrant methylation progression with cancer for acquiring driving mutations suggested by Brocks et al. in their study of prostate cancer evolution [34]. Future study on monitoring cancer status with methylation based biomarkers could validate this hypothesis.

### Considering heterogeneity in methylation measurement

In comparison of RRBS and qMSP, PENK related methylation measured with a shorter region by qMSP appeared to have stronger correlation with BCs suggesting gene methylation level could be better measured rather than adopting a simple mixture of methylation proportion of all related loci. Mikeska et al. concluded that heterogeneity should be taken into account in measuring methylation [35]. Scherer et al. further concluded and compared serval scores in quantifying within-sample methylation heterogeneity [36]. Applying appropriate quantification methods with RRBS or targeted bisulfite sequencing should improve BC detection accuracy. However, clinical application of sequencing techniques is mainly limited by the requirements of expensive instruments and reagents and a multidisciplinary operator team.

## Conclusion

In conclusion, we developed a three-gene methylation panel P3 for BC detection with qMSP, which demonstrated performance improvement in a cohort with 207 samples and was proven to be stable in analytical validation. Although further validation will be required for commercialising the panel on qMSP platform, it has shown great potential value in clinical application.

## Supplementary Information

**Additional file 1.**

## Data Availability

Sequencing data was deposited to NCBI SRA database and can be accessed via https://www.ncbi.nlm.nih.gov/sra/PRJNA715028. Differential methylation sites and qMSP results can be found in supplementary materials.

## Abbreviations

ANOVA: Analysis of variance
AUC: Area under the curve
BC: Bladder cancer
DMR: Differentially methylated region
DMS: Differential methylation site
DNA: Deoxyribonucleic acid
FISH: Fluorescence in situ hybridization
IVD: In vitro diagnostics
MIBC: Muscle invasive bladder cancer
MS-MLPA: Methylation-Specific Multiplex Ligation-Dependent Probe Amplification
NMIBC: Non-muscle invasive bladder cancer
NMP-22: Nuclear matrix protein 22
PCR: Polymerase Chain Reaction
qMSP: Quantitative methylation-specific PCR
ROC: Receiver Operating Characteristic
RRBS: Reduced representation bisulfite sequencing
SVM: Support vector machine
